# Neural correlates of religious behavior related to Christianity: an ALE meta-analysis

**DOI:** 10.3389/fpsyg.2025.1557796

**Published:** 2025-03-07

**Authors:** Andy Wai Kan Yeung, Natalie Sui Miu Wong, Ice S. Y. Tsui, Terence C. P. Lee

**Affiliations:** ^1^Oral and Maxillofacial Radiology, Applied Oral Sciences and Community Dental Care, Faculty of Dentistry, The University of Hong Kong, Hong Kong, Hong Kong SAR, China; ^2^Oral and Maxillofacial Surgery, Faculty of Dentistry, The University of Hong Kong, Hong Kong, Hong Kong SAR, China; ^3^Research Centre for Environment and Human Health, School of Continuing Education, Hong Kong Baptist University, Kowloon, Hong Kong SAR, China; ^4^College of International Education, Hong Kong Baptist University, Kowloon, Hong Kong SAR, China

**Keywords:** fMRI, CBMA, activation likelihood estimation, meta-analysis, Christianity, religiosity

## Abstract

**Background:**

Multiple neuroimaging studies have been published to report brain processing of religious behavior related to Christianity, such as prayer and recitation of the Bible. This meta-analysis aimed to pool data across studies to identify brain regions consistently activated in response to such religious tasks.

**Methods:**

Web of Science, Scopus, and PubMed were queried to identify relevant studies. Brain coordinates and sample size were manually extracted from the identified studies, and entered into a dedicated software called GingerALE to conduct meta-analysis.

**Results:**

Meta-analytic results based on 11 studies showed that brain processing of Christian behavior was associated with the right middle frontal gyrus and superior frontal gyrus, with a peak location (at 44, 38, 26; cluster size = 760 mm^3^) preferentially associated with working memory, cognitive task, and executive function according to Neurosynth data. Sub-analyses on Christian subject data revealed no significant results at the pre-defined threshold. With a more liberal threshold, Christian tasks > non-Christian tasks showed activation in the anterior cingulate and medial frontal gyrus (peak at 4, 48, −4; cluster size = 256 mm^3^) that were frequently associated with reward, self-referential, and reinforcement learning, whereas non-Christian tasks > Christian tasks showed activation in the right middle frontal gyrus (peak at 48, 36, 24; cluster size = 472 mm^3^) that frequently associated with working memory, executive function, arithmetic, and calculation.

**Conclusion:**

This study has revealed the relevance of frontal and limbic regions to Christian behavior.

## 1 Introduction

The intersection of religion and neuroscience has emerged as a popular domain of research study, offering insights into how religious experiences are represented in the brain. Religious behavior can be both external and internal, voluntary (proactive) or unconscious (reactive). Christian practices such as reciting the Bible and engaging in active prayer constitute complex experiences that encompass cognitive, emotional, and spiritual dimensions. In particular, prayer can be divided into learned prayer (pre-formulated, e.g., Lord’s Prayer) and spontaneous prayer (i.e., personalized). Understanding the neural mechanisms underlying these practices can reveal the cerebral processing of religious beliefs and spiritual activities. For example, Newberg et al. (2006) investigated the neural correlates of glossolalia (speaking in tongues) among Christians. Compared to singing with gospel music, glossolalia caused decreased activity in the prefrontal cortices, left caudate and left temporal pole, and increased activity in the left superior parietal lobe and right amygdala. The authors interpreted the findings as a sign of reduced intentional control and altered emotional activity. Meanwhile, Schjødt et al. (2008) has reported that Christian subjects showed a heightened activation at the caudate, an important part of the dopaminergic reward system, when they performed the Lord’s Prayer and personal prayer. In contrast, the caudate was deactivated when they performed a rhyme or made a wish to Santa Claus.

The effect of long-term religious exercise was also investigated. For instance, a week of spiritual retreat based on the Spiritual Exercises of St. Ignatius of Loyola was found to significantly reduce the transporter binding of dopamine in basal ganglia and of serotonin in the midbrain, altering resting-state functional connectivity between the posterior cingulate, frontal and parietal regions that form a network in the brain associated with religious or spiritual processing, as well as reducing the self-perceived levels of tension and fatigue (Newberg et al., 2018; Wintering et al., 2020). Results from the abovementioned examples were reasonable, as Christian experiences often involve personal reflection, emotional engagement, and a sense of connection with the divine. These findings suggest that engagement in religious activities activates brain regions associated with self-referential cognition and reward processing.

Neuroimaging techniques such as functional magnetic resonance imaging (fMRI) and, to a lesser extent, positron emission tomography (PET), have been pivotal in mapping brain activities associated with various religious tasks. Studies could utilize these techniques to explore the neural correlates of religious practices such as scripture recitation, prayer, and meditation. While individual studies have provided valuable insights, results were often inconsistent due to heterogeneity in study design and subject characteristics. For example, a study on Carmelite nuns who lived in secluded areas and separated from the society requested them to recall most intense mystical experience as a Carmelite (Beauregard and Paquette, 2006). The task was very personal as the recall from each nun would be unique, so that the results might be less generalizable than those synthesized from more impersonal tasks such as reciting scripture repeatedly.

Given the paradigm constraints unfolded above, embracing a meta-analytic approach allows for a more robust understanding by pooling data across studies, therefore increasing the statistical power and generalizability of the findings (Müller et al., 2018; Yeung, 2023). In the current meta-analysis, we attempted to synthesize existing neuroimaging research in order to identify the neural correlates of Christian experiences. As religious behaviors often provide emotional satisfaction and involve deep personal reflection as elaborated from the abovementioned examples, our central hypothesis was that the pooled data from neuroimaging studies on Christian behaviors (tasks) would show significant brain activation in regions responsible for reward processing and self-referential cognition.

## 2 Materials and methods

### 2.1 Literature search and screening

Published studies that reported neuroimaging results on Christian behaviors would be initially eligible in this work. Adhering to the preferred reporting items for systematic reviews and meta-analyses (PRISMA) guideline, papers were searched from multiple literature databases, namely Web of Science, Scopus, and PubMed, on 28 August 2024. No review protocol was preregistered. The following search string was used to search for the titles, abstracts and keywords of papers: (fMRI OR “functional magnetic resonance imaging” OR “functional MRI” OR sMRI OR “structural magnetic resonance imaging” OR “structural MRI” OR “functional neuroimaging” OR “structural neuroimaging”) AND (christian* OR jesus OR bible OR biblical OR pray*). Since PubMed did not record keywords of papers, only the title and abstract fields were searched in this database. No filter was placed on publication year, meaning that existing articles on the topic published up to the search date could be included. Reference lists of relevant publications were manually screened to identify potentially missed papers. This study did not involve human or animal subjects and hence ethical approval was not applicable.

**Figure 1:**
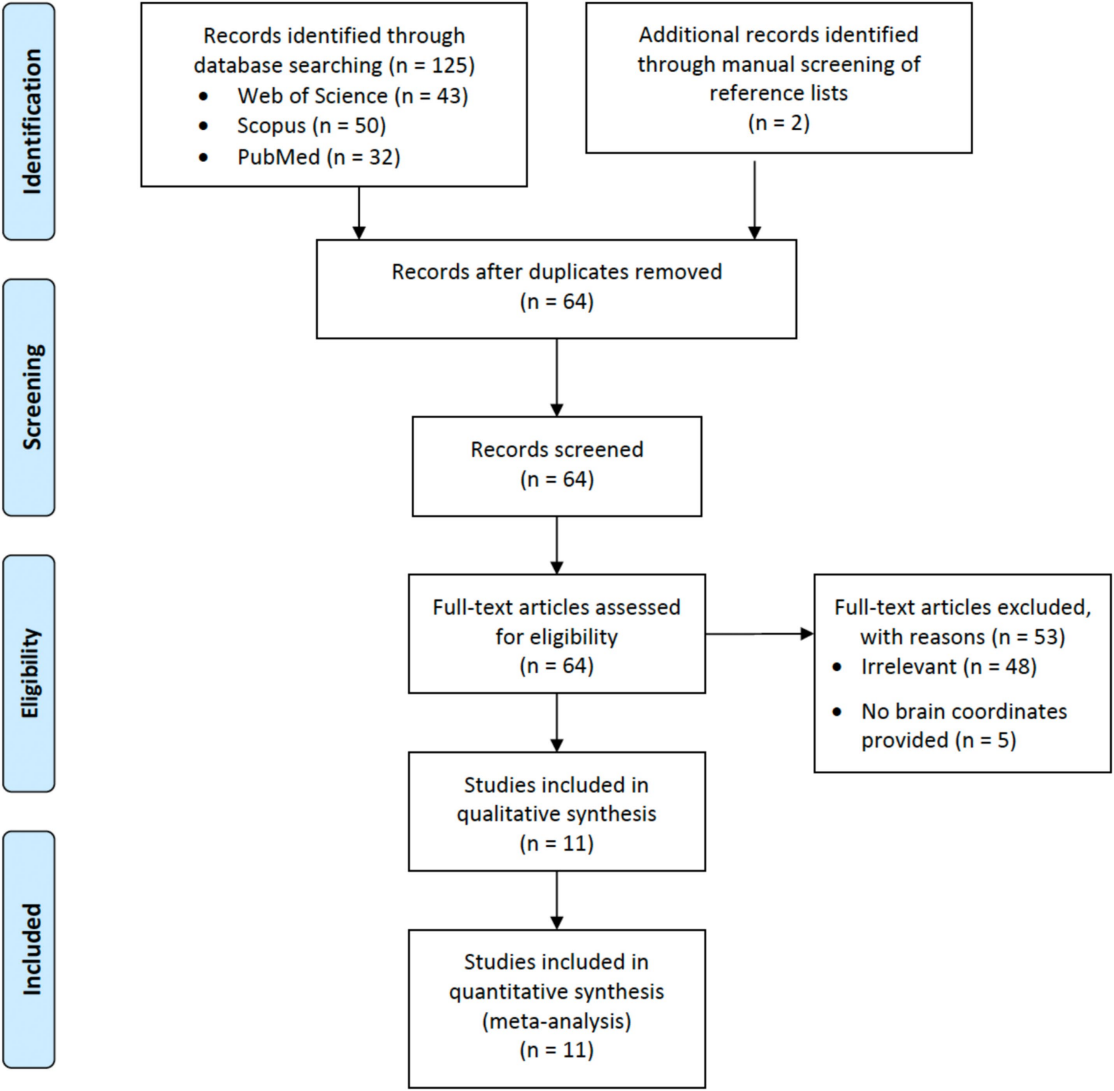
Flow diagram for literature search.

The search initially yielded 127 papers. After excluding duplicates, 64 remained. Forty-eight of them were excluded after further screening because of irrelevance, while another five were excluded because they did not provide brain coordinates for meta-analysis. The list of excluded studies is provided in Supplementary File 1. The corresponding authors of these papers were contacted to request for the brain coordinates, but no response was received. Finally, 11 studies remained (Figure 1).

### 2.2 Information recorded from the analyzed papers

The following items were manually extracted from the analyzed papers, including the number of subjects, their age and gender, the religious status of the subjects, the contrasts selected for brain coordinate extraction, the number of brain coordinates extracted, as well as the statistical threshold used to report fMRI results (the most stringent one was recorded if there were multiple items) (Table 1).

**Table 1:** Details of the 11 meta-analyzed papers.

Study	Journal	No. of subjects	Mean age (SD)	Religious status	Subject type: contrast selected	No of brain coordinates extracted	Statistical threshold
Azari et al. (2001)	Eur J Neurosci	12 (8M, 4F)	28.5 (3.9)	6 Free Evangelical Fundamentalists (self-identified, members of the Christian community) vs. 6 non-Christians	Christian: Recite Psalm 23:1 > Recite a well-known nursery rhyme	4	Voxel *P*_unc_ < 0.001
Beauregard and Paquette (2006)	Neurosci Lett	15 (15F)	49.9 (11.3)	15 Carmelite nuns	Christian: Recall most intense mystical experience as a Carmelite > Recall most intense state of union with another human (and vice versa)	7	Voxel *P*_unc_ < 0.001 + cluster >10 voxels
Han et al. (2008)	Soc Neurosci	28 (14 M, 14F)	23.1 (4.6)	14 Christians (self-identified, members of faith community) vs. 14 non-Christians	Christian: Judge if the selected adjectives correctly describe Jesus > Judge oneself (and vice versa) Non-Christian: Judge oneself > Judge Jesus	3	Voxel P_unc_ < 0.01
Harris et al. (2009)	PLOS One	30 (14M, 16F)	21.8 (Ranged 18–30)	15 Christians vs. 15 non-Christians (self-identified)	All: Judge true or false for statements about Christianity > Judge for non-religious statements (and vice versa) Non-Christian > Christian: True statement > False statement	35	Cluster *P*_corr_ < 0.05, cluster-forming threshold *P*_unc_ < 0.01
Ge et al. (2009)	Hum Brain Mapp	28 (14M, 14F)	23.1 (4.6)	14 Christians (self-identified, members of faith community) vs. 14 non-Christians	Non-Christian: Judge if the selected adjectives correctly describe Jesus (increased covariation w/vmPFC activity during Judge Zhu Rongji (a government leader) > Judge oneself)	4	Cluster *P*_corr_ < 0.05
Schjødt et al. (2009)	Soc Cogn Affect Neurosci	20 (6 M, 14F)	25.4 (Ranged 21–32)	20 Christians from Danish Inner Mission (church members)	Christian: Personal praying > Make wishes to Santa Claus (and vice versa)	12	Voxel *P*_FDR_ < 0.05 + cluster >15 voxels
Schjødt et al. (2011)	Soc Cogn Affect Neurosci	36 (14 M, 22F)	24.7 (4.2)	18 Christians mainly from Pentecostal Movement (church members) vs. 18 non-Christians	Christian: Listen to intercessory prayers from a non-Christian > Listen to intercessory prayers from a Christian known for his healing powers	17	Voxel *P*_FDR_ < 0.05 + cluster >15 voxels
Neubauer (2014)	Relig Brain Behav	14 (6 M, 8F)	34 (Ranged 19–62)	14 Renewalists (members of prayer groups and churches)	Christian: Active personal prayer > speak to a loved one (and vice versa)	4	Cluster *P*_corr_ < 0.05, cluster-forming threshold *P*_unc_ < 0.01
Silveira et al. (2015)	PsyCh J	1 (1 M)	72	1 Catholic bishop	Christian: Judge if agree or disagree with statements from Daodejing (non-religious) > Psalm	4	Voxel *P*_FWE_ < 0.05
Elmholdt et al. (2017)	Front Hum Neurosci	28 (12 M, 16F)	24 (Ranged 21–32)	28 Protestants from Danish Lutheran Church (church members)	Christian: Pray to an imaginary Mr. Hansen during induced pain > Pray to God during induced pain (personal prayer)	8	Cluster *P*_FWE_ < 0.05
Kober et al. (2017)	Front Hum Neurosci	40 (18 M, 22F)	23.9 (4.8)	40 self-identified Christians (27 Catholics +6 Protestants +7 seceded from Church)	Christian: GMV correlated to neurofeedback training performance, 3 contrasts of: positive correlation (seldom prayed group), negative correlation (seldom prayed group), and negative correlation (frequently prayed group); collapsed into 2 contrasts based on grouping	3	Cluster *P*_FWE_ < 0.05, cluster-forming threshold *P*_unc_ < 0.001
Total		252 (107 M, 145F)				101	

P_corr_, *P*-value corrected for multiple comparisons with unspecified method. P_FDR_, P corrected for false-discovery rate. P_FWE_, P corrected for familywise-error rate. P_unc_, P uncorrected for multiple comparisons.

### 2.3 ALE meta-analysis

Using the ALE method, the coordinate-based meta-analysis converged brain coordinates and sample size data across the included papers in order to identify the brain regions consistently activated by means of probability distribution modeling (Eickhoff et al., 2009). From each included paper, the brain coordinates of regions with significant activation were extracted. Within a paper, if there were multiple similar contrasts tested and reported, the most representative ones would be selected. For example, a study made comparisons among three conditions: active prayer, speaking to the loved one (imagining a close personal friend), and baseline (imaging a variety of animals while naming them consecutively) (Neubauer, 2014). In this case, the brain coordinates from the contrasts of prayer > loved one and loved one > prayer were extracted, whereas those from the contrasts involving baseline were discarded.

A minority of the included papers reported brain coordinates in Talairach system. To unify the format, these coordinates were converted into the MNI system by Lancaster transform (Lancaster et al., 2007). The conversion was performed with the ALE meta-analytic software GingerALE 3.0.2 (freely available from http://brainmap.org/ale/) (Eickhoff et al., 2012; Eickhoff et al., 2009; Turkeltaub et al., 2012). Subject-based full-width half-maximum values were applied with default settings (Eickhoff et al., 2009). The more conservative whole-brain mask, but not the dilated one, was selected. Following the latest recommendations of using GingerALE for ALE meta-analysis (Eickhoff et al., 2016; Müller et al., 2018; Yeung et al., 2023; Yeung et al., 2019), a cluster of brain voxels was considered significantly activated if it had a cluster *p* < 0.05 (corrected by familywise error rate, P_FWE_) with a primary cluster-defining threshold of *p* < 0.001. If no significant result was found for some of the meta-analyses, exploratory analyses were conducted with a more liberal threshold of voxel *p* < 0.001 (uncorrected for multiple comparisons, P_unc_) and cluster size > 200 voxels. Thresholded ALE maps were overlaid onto the Colin brain template in MNI space (Holmes et al., 1998) for results visualization with Mango 4.0 (freely available from http://ric.uthscsa.edu/mango/mango.html).

Three ALE meta-analyses were conducted: (1) an overall meta-analysis that covered all data extracted from all included studies (19 contrasts, 101 brain coordinates), (2) a meta-analysis that covered data from Christian tasks > non-Christian tasks performed by Christian subjects (5 contrasts, 17 brain coordinates), and (3) a meta-analysis that covered data from non-Christian tasks > Christian tasks performed by Christian subjects (7 contrasts, 41 brain coordinates). Due to the small number of studies adopted, similar meta-analyses based on data from non-Christian subjects, or, a meta-analysis directly comparing Christian and non-Christian subjects could not be performed.

### 2.4 Location-based analysis by Neurosynth

The peak coordinates resulted from the ALE meta-analyses were entered into the Neurosynth database (freely available from: https://neurosynth.org/) (Yarkoni et al., 2011) to conduct its in-built location-based analysis. This analysis provides associations between activation at the pre-defined voxel and terms indexed by Neurosynth. The location-based analysis has four output metrics, namely z-score, posterior probability, functional connectivity (r), and meta-analytic coactivation (r), all of which are automatically computed based on data hosted by Neurosynth. The first metric, z-score, is obtained from a two-way ANOVA testing for the presence of a non-zero association between term use and voxel activation. In other words, the magnitude of the z-score informs researchers of whether activation in a region occurs more consistently for studies that mention a particular term than for studies that do not mention it. For the definitions of the other metrics, readers may refer to the FAQs section of Neurosynth database.

## 3 Results

### 3.1 Study characteristics

There were 11 papers included in the current meta-analysis, published in the period between 2001 and 2017 in journals with an impact factor issued by Journal Citation Reports 2023 (IF). Social Cognitive and Affective Neuroscience (IF 3.9) and Frontiers in Human Neuroscience (IF 2.4) each published two papers adopted in the meta-analysis. The number of subjects per study ranged from 12 to 40, with the exception of a single-subject study (Table 1). The mean age of the subjects reported from the papers fell within the range of 21.8–72 years. Most of them recruited subjects with a balanced gender ratio. Six studies recruited Christian subjects only, while the Christian subjects came from a variety of Christian denominations. The studies mainly reported significant results for religious task > secular task and vice versa, from Christian subjects. The studies mainly used fMRI as their imaging modality, except for one PET study (Azari et al., 2001) and one structural MRI study (Kober et al., 2017). The extracted brain coordinates are provided in Supplementary File 2.

### 3.2 ALE meta-analysis: all data

**Figure 2:**
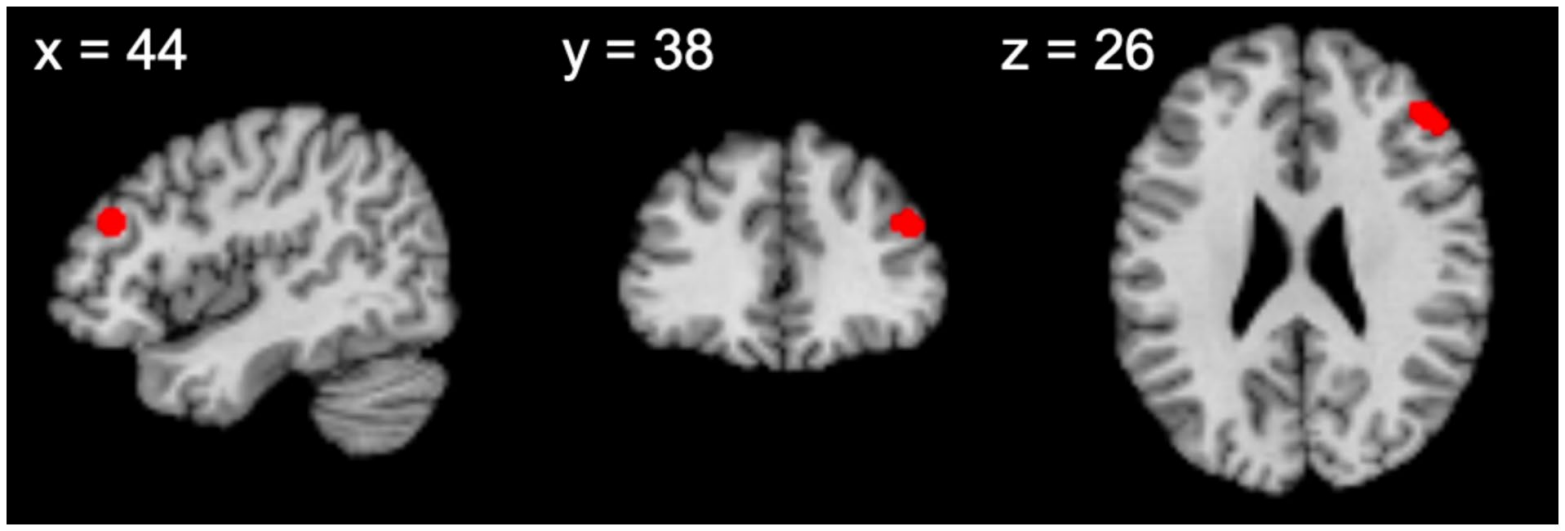
Significant activation from the overall analysis (cluster *P*_FWE_ < 0.05 and cluster-forming threshold of *p* < 0.001).

There was one significant cluster that mainly covered the right middle frontal gyrus as well as the superior frontal gyrus (Brodmann area 9) (Figure 2 and Table 2). Location-based analysis suggested that this location is frequently associated with working memory, cognitive task, and executive function (Table 3).

**Table 2:** Meta-analytic results of papers that reported religious behavior related to Christianity.

Cluster no.	Cluster size (mm^3^)	Side	Brain region	Peak voxel brain coordinate			ALE value (×10^−2^)
				*x*	*y*	*z*	
Overall analysis							
1	760	R	Middle frontal gyrus	44	38	26	1.57
Christians: Christian task > non-Christian task (exploratory analysis)^a^							
1	256	R	Anterior cingulate	4	48	−4	0.75
Christians: non-Christian task > Christian task (exploratory analysis)^a^							
1	472	R	Middle frontal gyrus	48	36	24	1.25

Brain coordinates are reported in Montreal Neurological Institute standard. ^a^ Exploratory analysis results were reported at *P*_unc_ < 0.001 + cluster size > 200 voxels.

**Table 3:** Representative associations of the peak coordinates with meta-analysis maps hosted by Neurosynth.

Concept/theme	*z*-score	Posterior probability	Functional connectivity (*r*)	Meta-analytic coactivation (*r*)
Cluster 1 from overall analysis (peak at 44, 38, 26)				
Working memory	10.07	0.74	0.44	0.63
Cognitive task	6.11	0.8	0.01	0.04
Executive	5.95	0.69	0.23	0.32
Cluster 1 from Christians: Christian task > non-Christian task (peak at 4, 48, −4)				
Reward	6.6	0.72	0.21	0.21
Self-referential	4.29	0.76	0.28	0.32
Reinforcement learning	4.24	0.81	0.06	0.1
Cluster 1 from Christians: non-Christian task > Christian task (peak at 48, 36, 24)				
Working memory	7.52	0.71	0.46	0.62
Executive	6.57	0.71	0.2	0.27
Calculation	5.01	0.81	0.3	0.36
Arithmetic	4.68	0.79	0.17	0.24

### 3.3 ALE meta-analysis: Christian tasks > non-Christian tasks (Christian subjects)

**Figure 3:**
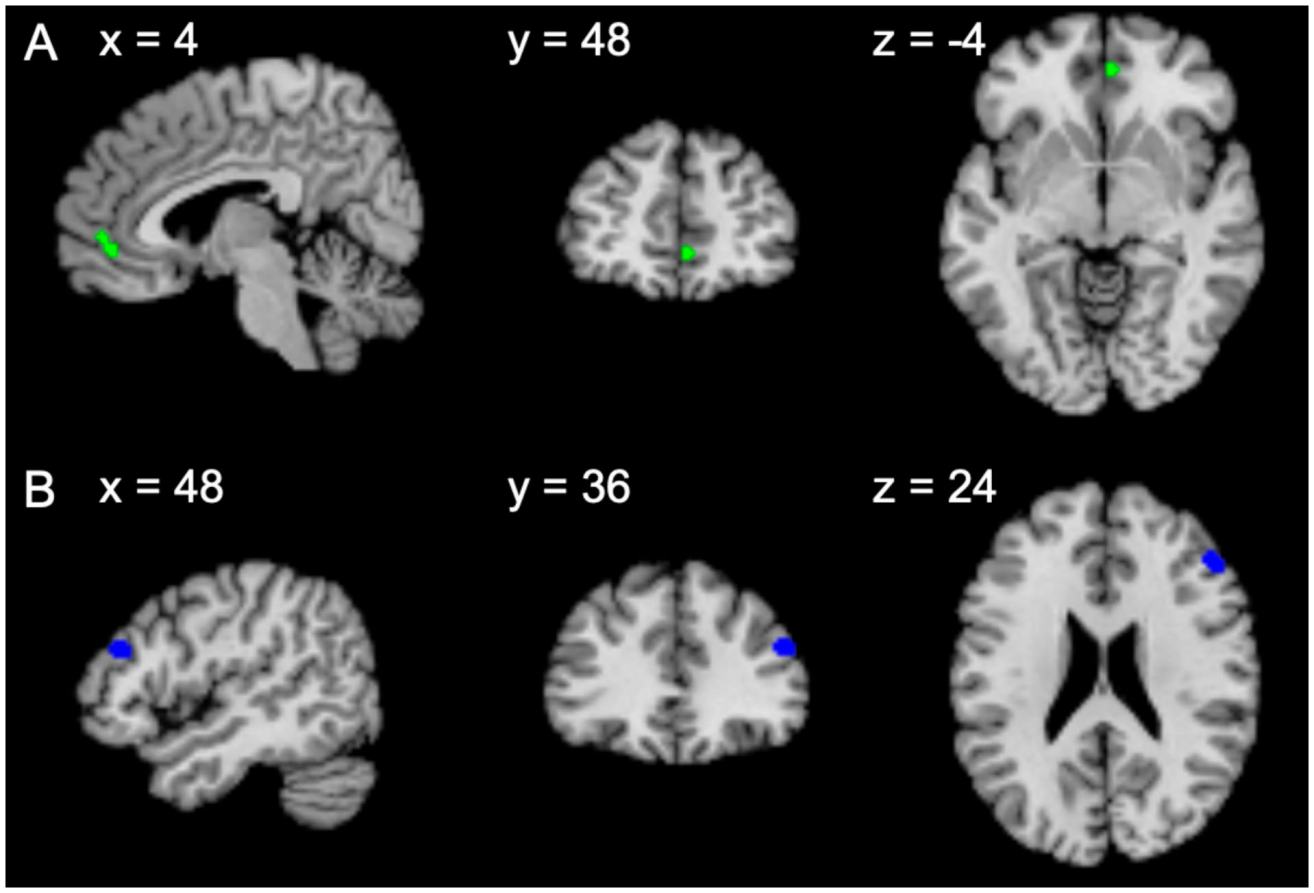
Significant activation from the exploratory analyses (voxel P_unc_ < 0.001 and cluster size >200 voxels) of Christian subjects on **(A)** Christian task > non-Christian task, and **(B)** non-Christian task > Christian task.

Under the planned threshold, no significant cluster was found. Exploratory analysis revealed one significant cluster in the anterior cingulate that extended to the medial frontal gyrus (mainly Brodmann area 32, also Brodmann area 10) (Figure 3A and Table 2). Location-based analysis suggested that this location is frequently associated with reward, self-referential, and reinforcement learning (Table 3).

### 3.4 ALE meta-analysis: non-Christian tasks > Christian tasks (Christian subjects)

Under the planned threshold, no significant cluster was found. Exploratory analysis showed one significant cluster that covered the right middle frontal gyrus (Brodmann area 9) (Figure 3B and Table 2). Location-based analysis suggested that this location is frequently associated with working memory, executive function, arithmetic, and calculation (Table 3).

## 4 Discussion

This neuroimaging meta-analysis has revealed that the processing of Christian belief could be associated with the right middle frontal gyrus and the superior frontal gyrus, with a peak location preferentially associated with working memory, cognitive task, and executive function according to existing literature. Sub-analyses on Christian subject data revealed no significant results at the pre-defined threshold. With a more liberal threshold, Christian tasks > non-Christian tasks showed activation in the anterior cingulate and medial frontal gyrus, which are frequently associated with reward, self-referential, and reinforcement learning, whereas non-Christian tasks > Christian tasks showed activation in the right middle frontal gyrus, which is frequently associated with working memory, executive function, arithmetic, and calculation. Readers can refer to comprehensive review papers for more complete understanding or a framework regarding neural processing of religion and spirituality (Grafman et al., 2020; Van Elk and Aleman, 2017). For example, it was discussed that the anterior cingulate is involved in acquiring and maintaining intuitive supernatural beliefs, as well as detecting conflicts between religious beliefs and task stimuli or demands (Grafman et al., 2020; Van Elk and Aleman, 2017).

From a theological standpoint, particularly within Protestant traditions, the neural correlates from the middle frontal gyrus and superior frontal gyrus may underscore the integral role of cognition in the relationship between the self and the Transcendent. The activation of brain areas linked to working memory, cognitive tasks, and executive function aligns with the Reformation principle of “Sola Scriptura” (Engelder, 1916), emphasizing the importance of the Word in divine revelation. This cognitive engagement reflects the belief that God’s re-connection with humanity is mediated through intellectual recognition and understanding of the Scriptures. Dialectical theology, as postulated by Barth (1933), posits that divine revelation is external and revelatory, implying that cognitive faculties are essential for receiving and interpreting this revelation. In the same vein, Pannenberg’s narrative and evolutionary theology (Pannenberg, 1968) posits that divine revelation is an ongoing, historical process that requires active cognitive participation to understand the unfolding narrative of God’s Spirit throughout history. These theological perspectives collectively highlight that cognitive appraisal and therefore neurological engagement are central to the experience and practice of Christian faith, as evidenced by the neural correlates identified in this study. The theological thoughts mentioned here are merely examples. In this research context, exploring how theological insights align with current and future neurological findings could promote a productive line of study.

A recent neuroimaging meta-analysis of 22 fMRI studies on Buddhist meditation found no significant results at false-discovery rate-corrected *p* < 0.05, or *P*_FDR_ < 0.05 (Chaudhary et al., 2023). At *P*_unc_ < 0.0001, activation was found at the medial frontal gyrus, precuneus, insula, and inferior parietal lobule, for which the authors considered to be associated with social cognition, performance monitoring, self-referential processing and self-awareness. Here, it could be observed that the current results and the results from Buddhist meditation shared one similarity: the activation of medial frontal gyrus, a part of the default mode network responsible for self-referential processing (Raichle et al., 2001).

In the current meta-analysis, all included studies recruited generally healthy subjects. In fact, the neural correlates of prayer have been elucidated in patients as well. For example, a 6-week intervention with a Christian form of prayer that “that focused on forgiveness and psycho-spiritual healing” was found to increase the activation in the medial prefrontal cortex when focusing on traumatic memory among patients with depression (Baldwin et al., 2016). Meanwhile, craving following prayer (unclear if it was Christian prayer or not) negatively correlated with activation in the default mode network and brain regions associated with self-referential processing, among people with alcohol-use disorders who joined Alcoholics Anonymous (Galanter et al., 2017).

One possible common confounding factor shared by the neuroimaging studies on prayer (praying or listening to prayer) is the environmental factor. It is very different (and uncomfortable) to pray inside the narrow space of an MRI machine with loud noise in supine position compared to sitting in a chair, lowering the head and praying in a silent environment (Ladd et al., 2015). In a previous study (Ladd et al., 2015), subjects with diverse religious backgrounds (including agnostic and atheist) were asked to pray in a silent room and inside an fMRI machine with the operating noise (without a real scan). The subjects indicated that prayer in the fMRI setting was significantly less similar to their normal prayer experience, less comfortable, and they were less able to focus on praying compared to prayer in the silent room.

In fact, the experimental limitations of inducing religiosity during fMRI scans have been addressed by some of the included studies in the current meta-analysis. For example, some Christian subjects commented that to induce repeated, transient religious states within a single scanning session was antithetical and disrespectful to authentic religious experience; instead, they agreed to induce and sustain the religious state for the entire duration of a scanning session by reciting Psalm 23:1 (Azari et al., 2001). In another study, some Carmelite nuns commented that “God cannot be summoned at will” so that they agreed to recall a mystical experience rather than actually attempting to achieve one, during fMRI scan (Beauregard and Paquette, 2006).

Another issue that could be identified from the meta-analyzed studies is the lack of data from inter-group comparison between Christian and non-Christian subjects. Many studies either reported insignificance, or simply did not report such results. Other studies recruited only Christian subjects, so that such comparison was not possible. While it is insightful to reveal brain regions with significant activations during religious state experienced by Christian subjects, a more comprehensive understanding of the neurobiology could be obtained by comparing and contrasting the similarities and differences between Christian and non-Christian subjects. However, dividing people into Christian vs. non-Christian may raise methodological/theological concerns in this research context. With more data accumulated in the future, a more interesting analysis would be to examine which brain regions show activation levels highly correlated to certain measures of religiosity or spirituality.

It could be argued that some of the earlier fMRI studies on Christianity were underpowered. Since the analysis of fMRI data involves comparisons between thousands of brain voxels, it is critical to report results that are controlled for the false positive rate through numerous means, such as FWE or FDR (Han et al., 2019; Poldrack et al., 2017). It is reassuring to see that the meta-analyzed studies published since 2009 have adopted statistical thresholds corrected for multiple comparisons. Future studies are encouraged to continue with the practice of reporting corrected results, with a possible addition of providing unthresholded brain maps so that readers may gain access to a more complete dataset (Poldrack et al., 2017).

Finally, this meta-analysis has some limitations. First, the subjects were mainly young and middle-aged adults, so that the results may not be generalized to children and adolescents. The analyzed studies usually have a small sample size. Moreover, very few original studies were identified, rendering it impossible to acquire enough data to directly draw comparison between Christian and non-Christian subjects. Again, even though the current work has reported sub-analyses on the differences between Christian and non-Christian tasks, readers should be reminded of their exploratory nature. It is because the small number of experiments per group did not meet the recommended number of 17–20 (Eickhoff et al., 2016) and no significant results were found when the recommended statistical threshold with FWE correction was adopted (Müller et al., 2018). Besides, the experimental designs of the included studies were heterogeneous, and therefore it was not possible to compare the effect between formal written prayer and informal personal prayer. With more studies published in the future, perhaps it would be interesting to investigate whether there exists different neural correlates of recitation versus meditation, and whether belief and practice (behavior) can enhance each other through a positive feedback loop, as beliefs and believing are different concepts (Seitz, 2022).

## 5 Conclusion

Within the limitations of this meta-analysis, it was concluded that the analyzed Christian behavior could be associated with the right middle frontal gyrus and superior frontal gyrus, with a peak location preferentially associated with working memory, cognitive task, and executive function. With a more liberal threshold, Christian tasks > non-Christian tasks among Christian subjects showed activation in the anterior cingulate and medial frontal gyrus, areas that are frequently associated with reward, self-referential, and reinforcement learning.

## Data Availability

The original contributions presented in the study are included in the article/Supplementary material, further inquiries can be directed to the corresponding author/s.

## Supplementary material

SUPPLEMENTARY FILE 1 List of excluded studies.

SUPPLEMENTARY FILE 2 List of brain coordinates extracted from the included studies.
