# Supplementary material for: Neural correlates of religious behavior related to Christianity: an ALE meta-analysis
Source: Front Psychol. 2025 Mar 7;16:1557796. doi: 10.3389/fpsyg.2025.1557796 (PMC11926137; doi:10.3389/fpsyg.2025.1557796)
Supplement: SUPPLEMENTARY FILE 2 — List of brain coordinates extracted from the included studies. [file Table_2.DOCX]

// Reference=MNI

// Azari, 2001: recite Psalm 23:1 > recite a well-known nursery rhyme

// Subjects=6

24 50 34

7 10 68

14 -82 46

-2 -48 -20

// Reference=MNI

// Harris, 2009: (All) Non-Christian > Christian: True statement > False statement

// Subjects=30

2 40 34

14 16 0

16 14 -8

46 30 34

-48 36 22

-36 64 2

32 64 4

-42 -48 46

48 -48 46

// Reference=MNI

// Harris, 2009: (All) Judge true or false for statements about Christianity > Judge for non-religious statements

// Subjects=30

-2 -22 30

-10 -72 36

0 30 26

-32 56 8

30 60 10

-36 10 -4

34 12 -8

42 40 26

-32 -62 48

32 -54 38

-32 -56 40

32 -54 38

-16 20 0

-14 12 -6

-50 10 2

12 16 64

2 -24 6

2 -72 -12

// Reference=MNI

// Harris, 2009: (All) Judge for non-religious statements > Judge true or false for statements about Christianity

// Subjects=30

-4 22 -18

-20 34 52

-56 -6 -16

-26 -40 -14

-14 -52 4

-40 38 -16

-48 16 -34

-22 -10 -22

// Reference=MNI

// Schjødt, 2009: personal praying > make wishes to Santa Claus

// Subjects=20

-10 -60 32

-40 -54 24

-46 6 -40

-14 50 22

// Reference=MNI

// Schjødt, 2009: make wishes to Santa Claus > personal praying

// Subjects=20

46 38 26

-32 26 14

8 18 50

18 -70 8

-28 -74 38

8 -98 0

-12 -36 64

30 -70 38

// Reference=MNI

// Schjødt, 2011: Listen to prayers from a non-Christian > Listen to prayers from a Christian known for his healing powers

// Subjects=18

-32 58 8

44 -46 42

64 -18 -20

-32 20 -20

-24 -78 -28

-42 -52 32

12 -84 -24

-58 -24 -18

36 -2 -18

-48 -2 -10

10 -54 48

-56 18 -2

-48 -24 18

48 20 -20

44 -4 -34

32 22 -14

-8 -32 -48

// Reference=MNI

// Neubauer, 2014: Active prayer > speak to a loved one

// Subjects=14

8 -6 58

-38 18 -6

// Reference=MNI

// Neubauer, 2014: speak to a loved one > Active prayer

// Subjects=14

6 56 -22

-2 -56 30

// Reference=MNI

// Silveira, 2015: Judge if agree or disagree with statements from Daodejing (non-religious) > Psalm

// Subjects=1

-42 14 24

-48 26 -6

-36 4 56

-62 -54 0

// Reference=MNI

// Elmholdt, 2017: pray to an imaginary Mr Hansen during induced pain > pray to God during induced pain

// Subjects=28

42 16 54

42 -58 50

50 34 24

42 50 -16

-38 -54 42

-52 -46 56

10 -72 44

-34 -72 54

// Reference=MNI

// Kober, 2017: GMV correlated to neurofeedback training performance (seldom prayed group)

// Subjects=20

30 25.5 -13.5

-30 -31.5 48

// Reference=MNI

// Kober, 2017: GMV correlated to neurofeedback training performance (frequently prayed group)

// Subjects=20

-7.5 60 -3

// Reference=TNT_MNIed

// Beauregard, 2006: Recall most intense mystical experience as a Carmelite > recall most intense state of union with another human

// Subjects=15

4.99 45.75 -24.89

-53.33 -54.56 42.06

51.68 -15.77 -19.59

-35.78 -52.52 62.85

5.31 51.44 1.44

3.09 47.72 -3.76

// Reference=TNT_MNIed

// Beauregard, 2006: Recall most intense state of union with another human > Recall most intense mystical experience as a Carmelite

// Subjects=15

-19.45 10.41 0.32

// Reference=TNT_MNIed

// Han, 2008: Judge if the selected adjectives correctly describe Jesus > Judge oneself

// Subjects=14

55.82 -62.68 41.01

// Reference=TNT_MNIed

// Han, 2008: Judge oneself > Judge if the selected adjectives correctly describe Jesus

// Subjects=14

-3.02 39.55 24.05

// Reference=TNT_MNIed

// Han, 2008: (Non-Christian) Judge oneself > Judge if the selected adjectives correctly describe Jesus

// Subjects=14

1 54.82 3.42

// Reference=TNT_MNIed

// Ge, 2009: Judge if the selected adjectives correctly describe Jesus (increased covariation w/ vmPFC activity during Judge Zhu Rongji (govt leader) > Judge self)

// Subjects=14

-11.16 -34.43 48.32

19.44 -42.68 73.26

43.06 -60.11 57.78

29.74 36.74 49.54
